# Changes of Photosynthetic Behaviors and Photoprotection during Cell Transformation and Astaxanthin Accumulation in *Haematococcus pluvialis* Grown Outdoors in Tubular Photobioreactors

**DOI:** 10.3390/ijms18010033

**Published:** 2016-12-26

**Authors:** Litao Zhang, Fang Su, Chunhui Zhang, Fengying Gong, Jianguo Liu

**Affiliations:** 1 Key Laboratory of Experimental Marine Biology, Institute of Oceanology, Chinese Academy of Sciences, Qingdao 266071, China; zhanglitao666@163.com (L.Z.); sufang116@126.com (F.S.); zhangchunhui13@mails.ucas.ac.cn (C.Z.); 18766216016@163.com (F.G.); 2 National-Local Joint Engineering Research Center for *Haematococcus pluvialis* and Astaxanthin, Yunnan Alphy Biotech Co., Ltd., Chuxiong 675012, China; 3 Laboratory for Marine Biology and Biotechnology, Qingdao National Laboratory for Marine Science and Technology, Qingdao 266071, China; 4 Institute of Oceanology, University of Chinese Academy of Sciences, Beijing 100049, China

**Keywords:** astaxanthin, chlorophyll *a* fluorescence transient, *Haematococcus pluvialis*, photoinhibtion, photosynthetic behavior

## Abstract

The cell transformation from green motile cells to non-motile cells and astaxanthin accumulation can be induced in the green alga *Haematococcus pluvialis* cultured outdoors. In the initial 3 d of incubation (cell transformation phase), light absorption and photosynthetic electron transport became more efficient. After five days of incubation (astaxanthin accumulation phase), the light absorption per active reaction center (ABS/RC) increased, but the efficiency of electron transport (*ψ_o_*) and the quantum yield of electron transport (*φ_Eo_*) decreased with increased time, indicating that the capacity of photosynthetic energy utilization decreased significantly during astaxanthin accumulation, leading to an imbalance between photosynthetic light absorption and energy utilization. It would inevitably aggravate photoinhibition under high light, e.g., at midday. However, the level of photoinhibition in *H. pluvialis* decreased as the incubation time increased, which is reflected by the fact that *F_v_*/*F_m_* determined at midday decreased significantly in the initial 3 d of incubation, but was affected very little after seven days of incubation, compared with that determined at predawn. This might be because the non-photochemical quenching, plastid terminal oxidase, photosystem I cyclic electron transport, defensive enzymes and the accumulated astaxanthin can protect cells against photoinhibition.

## 1. Introduction

*Haematococcus pluvialis*, a unicellular green alga, is widely known for its ability to accumulate large amounts of astaxanthin (3,3′-dihydroxy-β,β-carotene-4,4′-dione) [1,2,3]. The astaxanthin is used not only as a pigmentation source in aquaculture and poultry industries [4,5], but also has potential clinical applications due to its antioxidant activity, which is higher than that of β-carotene and α-tocopherol [6,7]. A two-step culture protocol is developed to cultivate *H. pluvialis* for astaxanthin production [8]. In the first step, favorable growth conditions are provided to accelerate *H. pluvialis* cell growth and to obtain a high biomass (cell growth phase); in the second step, stress conditions are provided to induce cell transformation from green motile cells to non-motile cells (cell transformation phase), and to induce the accumulation of astaxanthin (astaxanthin accumulation phase).

The cell transformation and astaxanthin accumulation are strongly light-induced processes for *H. pluvialis*, and can be regulated by photosynthetic redox status [9,10,11]. Therefore, understanding the changes of photosynthetic behaviors during the cell transformation and astaxanthin accumulation would provide some new insights into mechanisms of astaxanthin accumulation in *H. pluvialis*. However, most of the work published so far on *H. pluvialis* has been focused on the optimization of cell growth and the induction of astaxanthin accumulation of this alga [3,12]. Far less attention has been paid to the changes of photosynthetic behaviors, especially the energy fluxes of absorption, trapping and electron transport, in *H. pluvialis* during cell transformation and astaxanthin accumulation. Until recently, many works about the response of photosynthesis during cell transformation and astaxanthin accumulation in *H. pluvialis* grown indoors have been reported. Scibilia et al. studied the photosynthetic response to nitrogen starvation and high light in *H. pluvialis* [13]. Chekanov et al. investigated the modulation of photosynthetic activity in *H. pluvialis* during the incubation [14]. However, there is no work concerning outdoor photosynthetic response of this species reported.

The chlorophyll *a* fluorescence transient (labeled also as OJIP transient, because the fluorescence transient shows a polyphasic rise including phase O, J, I and P) combined with a JIP-test is one of the most popular tools in photosynthetic research [15,16,17,18]. It is possible to calculate several phenomenological and biophysical expressions of photosynthetic behaviors [19,20,21]. In the present study, we used the OJIP transient combined with the JIP-test to detect changes in photosynthetic behaviors, including the energy fluxes of absorption, trapping, and electron transport during the cell transformation and astaxanthin accumulation in *H. pluvialis* grown outdoors in tubular photobioreactors. Meanwhile, changes of photoprotective mechanisms during the cell transformation and astaxanthin accumulation were also analyzed. Determining the changes of photosynthetic behaviors and photoprotection during cell transformation and astaxanthin accumulation in *H. pluvialis* grown outdoors would help in optimizing astaxanthin production.

## 2. Results

**Figure 1:**
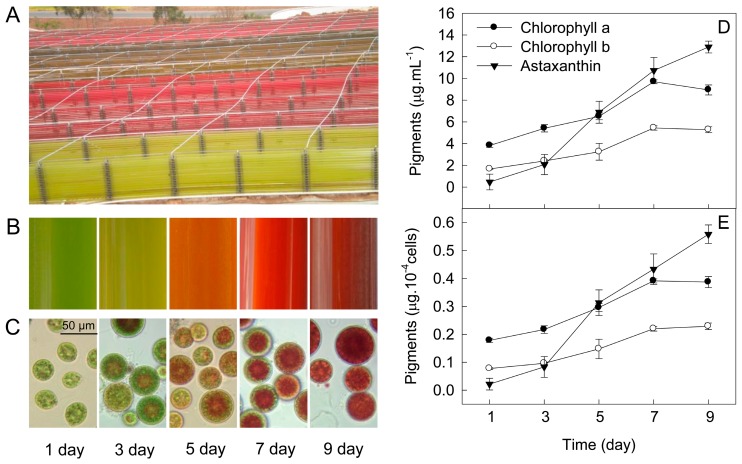
The processes of cell transformation and astaxanthin accumulation in *H. pluvialis* grown outdoors in tubular photobioreactors (**A**,**B**); The cell morphology during the cell transformation and astaxanthin accumulation in *H. pluvialis* in the tubular photobioreactors (**C**); The changes of chlorophyll and astaxanthin contents during cell transformation and astaxanthin accumulation in *H. pluvialis* cultured in tubular photobioreactors (**D**,**E**). Mean ± SE of five replicates are presented.

In tubular photobioreactors outdoors (Figure 1A,B), *H. pluvialis* cells underwent a morphological change over time (Figure 1B,C). The green motile cells lost their flagella and motility and then transformed into larger, non-motile cells during the first three days of incubation (cell transformation phase). Astaxanthin started to accumulate after three days of incubation (astaxanthin accumulation phase). The accumulation of astaxanthin in *H. pluvialis* was significant (*p* < 0.05) after five days of incubation (Figure 1C). The astaxanthin content increased from 0.47 μg·mL^−1^ (0.02 μg·10^4^ cells^−1^) to 12.89 μg·mL^−1^ (0.56 μg·10^4^ cells^−1^) during a nine-day incubation period (Figure 1D,E). The astaxanthin content on cellular dry weight increased from 0.11% to 0.97% (data not shown). Chlorophyll content increased gradually with time during the initial seven days of incubation, but decreased slightly after seven days of incubation in *H. pluvialis* (Figure 1D,E).

**Figure 2:**
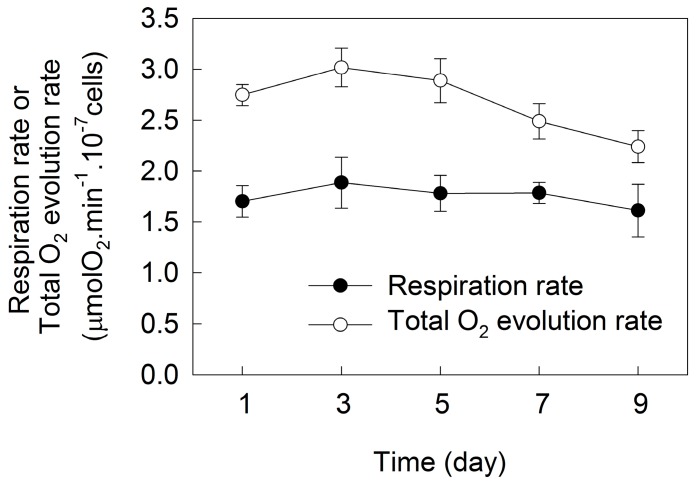
The respiration rate and total O_2_ evolution rate during cell transformation and astaxanthin accumulation in *H. pluvialis* cultured in tubular photobioreactors. Mean ± SE of five replicates are presented.

The respiration rate in *H. pluvialis* grown outdoors in tubular photobioreactors did not change during the incubation. The total O_2_ evolution rate, which is an indicator of photosynthetic capacity in plants, enhanced in the initial three days of incubation and decreased gradually over time after five days of incubation (Figure 2).

**Figure 3:**
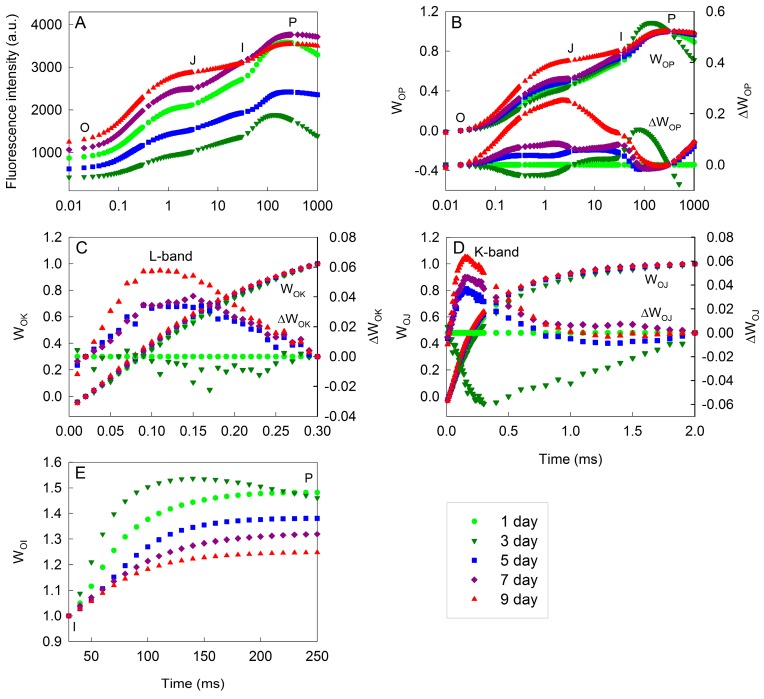
The chlorophyll *a* fluorescence (OJIP) transients during cell transformation and astaxanthin accumulation in *H. pluvialis* cultivated in tubular photobioreactors (**A**); the normalization of fluorescence transients between the O-P (**B**), O-K (**C**), O-J (**D**) and O-I (**E**) phases. The O, J, I, and P steps are marked in the plot. The corresponding difference kinetics between cells incubated for three, five, seven or nine days and cells incubated for one day (Δ*W* = *W* − *W*_(*cells incubated for 1 day*)_) are also depicted. Each transient represents the average of eight samples. The difference kinetics Δ*W_OJ_* and Δ*W_OK_* reveal the *K*-band (at about 300 μs) and *L*-band (at about 150 μs), respectively.

The changes of chlorophyll *a* fluorescence (OJIP) transients during the incubation in *H. pluvialis* cells are depicted (Figure 3A). The *H. pluvialis* cells showed a typical OJIP chlorophyll fluorescence transient (the O, J, I, and P steps are marked in the plot) at one day. The variation in the shape of OJIP transients was obvious with increased time during the incubation (Figure 3A), suggesting that photosystem (PS) II behaviors were changed during cell transformation and astaxanthin accumulation.

To clarify the detailed changes of PSII behaviors during the incubation in *H. pluvialis*, the changes in the phases of O-P, O-K, O-J and O-I in the relative variable fluorescence kinetics were analyzed by normalizing the OJIP transients (Figure 3B–E). It shows that fluorescence transients normalized between O and P were changed greatly with increased time during the incubation. The most distinct peak in the relative variable fluorescence kinetics was at the J-step (at about 2 ms), which increased with increased time after five days of incubation (Figure 3B), indicating that the ratio of Q_A_^−^/Q_A_ had increased [18,19]. In contrast, the J-step did not change in the first three days of incubation.

The *L*-bands did not change in the initial three days of incubation, and increased significantly (*p* < 0.05) over time after five days of incubation (Figure 3C). The higher *L*-band (Figure 3C) indicates a lower energetic connectivity of the PSII units, which results in a poorer utilization of the excitation energy and a lower stability of the system [17,18]. The *K*-bands (Figure 3D) decreased during the initial three days of incubation, and increased significantly (*p* < 0.05) after five days of incubation. The enhanced *K*-band indicates increased damaged to the donor side of PSII [15,17].

The maximal amplitude of fluorescence in the I-P phase of the OJIP transient reflects the pool size of end electron acceptors [17,18]. The maximal amplitudes of fluorescence in the I-P phase increased during the initial three days of incubation, and decreased significantly (*p* < 0.05) in *H. pluvialis* over time after five days of incubation (Figure 3E).

**Figure 4:**
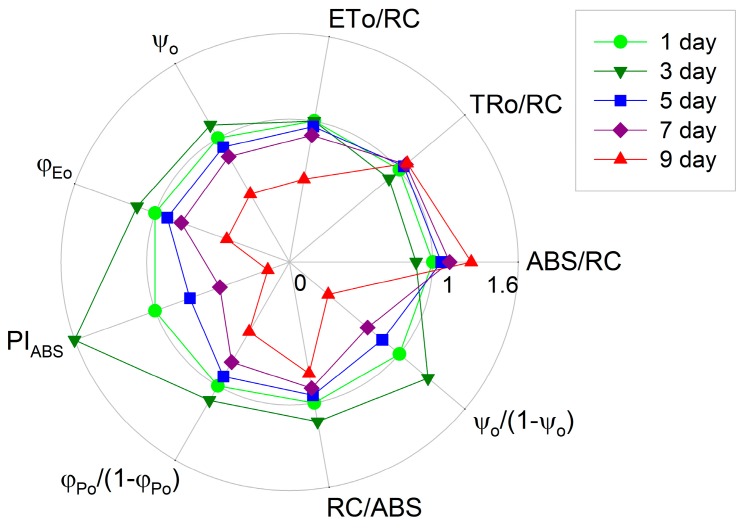
The light absorption per active reaction center (ABS/RC, the average antenna size), the trapping of excitation energy per active reaction center (TRo/RC), the electron transport per active reaction center (ETo/RC), the efficiency of electron transport (*ψ_o_*), the quantum yield of electron transport (*φ_Eo_*) and the performance index (PI_ABS_) as well as its individual partial components RC/ABS, *φ_Po_*/(1 − *φ_Po_*), and *ψ_o_*/(1 − *ψ_o_*), during cell transformation and astaxanthin accumulation in *H. pluvialis* grown in tubular photobioreactors. Each point represents the average of eight samples. All parameters in *H. pluvialis* cells incubated for one day were taken as one, whereas those in cells incubated for three, five, seven or nine days were expressed as a percentage of these parameters in cells incubated for one day. The original values of ABS/RC, TRo/RC, ETo/RC, *ψ_o_*, *φ_Eo_*, PI_ABS_, RC/ABS, *φ_Po_*/(1 − *φ_Po_*) and *ψ_o_*/(1 − *ψ_o_*) in the cells incubated for one day were 3.19 ± 0.02, 2.45 ± 0.02, 1.35 ± 0.04, 0.55 ± 0.02, 0.42 ± 0.01, 1.27 ± 0.03, 0.31 ± 0.00, 3.31 ± 0.17 and 1.23 ± 0.10, respectively.

The light absorption per active reaction center (ABS/RC, the average antenna size) and the trapping of excitation energy per active reaction center (TRo/RC) decreased slightly, the efficiency of electron transport (*ψ_o_*) and the quantum yield of electron transport (*φ_Eo_*) increased, and the electron transport per active reaction center (ETo/RC) did not change over time during the initial three days of incubation (Figure 4). This suggests that the capacities of light absorption and photosynthetic electron transport were highly efficient [17,22] in *H. pluvialis* during the first three days of incubation. However, the ABS/RC increased, whereas the ETo/RC, *ψ_o_*, *φ_Eo_* decreased, over time after five days of incubation. The ABS/RC increased about 26.7%, whereas the ETo/RC, *ψ_o_* and *φ_Eo_* decreased about 41.3%, 45.2% and 53.5%, respectively, during a nine-day incubation period.

The performance index PI_ABS_ is closely related to energy conservation capability and the activity of the photosynthetic apparatus [17,18,21]. Because it incorporates several parameters that are evaluated from the OJIP transient, it is the most sensitive indicator reflecting the photosynthetic performance. The PI_ABS_ in *H. pluvialis* increased significantly (*p* < 0.05) in the initial three days of incubation, and declined gradually over time after five days of incubation (Figure 4). The component parameters of PI_ABS_, the efficiency of absorption of light (RC/ABS), the performance due to the quantum efficiency of primary photochemistry (*φ_Po_*/(1 − *φ_Po_*)) and the performance due to the quantum efficiency of the conversion of excitation energy to electron transport (*ψ_o_*/(1 − *ψ_o_*)), increased significantly during the initial three days of incubation, and declined gradually over time after five days of incubation (Figure 4). The PI_ABS_ decreased by 84.0% in *H. pluvialis* during a nine-day incubation period. The individual effect on the component parameters of PI_ABS_ during a nine-day incubation period was as follows: RC/ABS decreased by 21.0%, *φ_Po_*/(1 − *φ_Po_*) decreased by 43.7%, and *ψ_o_*/(1 − *ψ_o_*) decreased by 64.7% (Figure 4). It seems that the contribution of the conversion of excitation energy to electron transport was the most sensitive partial component with increased time during the incubation of *H. pluvialis*.

**Figure 5:**
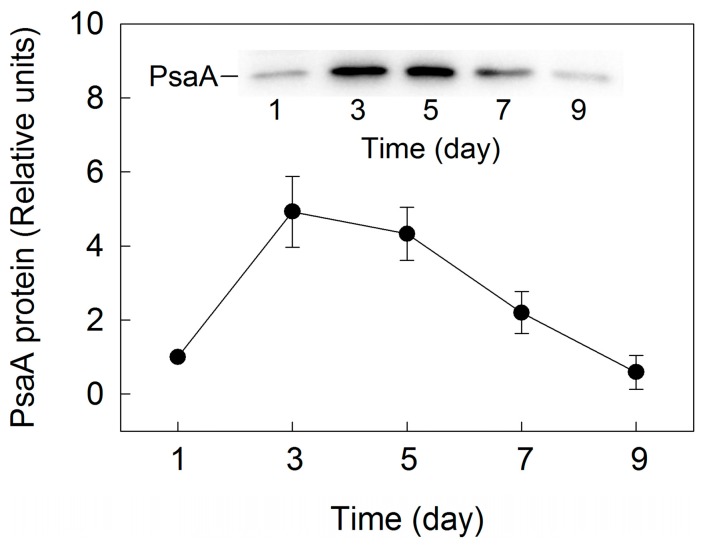
Densitometry of photosystem I core protein protein (PsaA) during cell transformation and astaxanthin accumulation in *H. pluvialis* cultured in tubular photobioreactors. Inset shows the levels of PsaA protein. Representative results of three independent replicates are shown.

To investigate the change of PSI activity during cell transformation and astaxanthin accumulation in *H. pluvialis* grown outdoors, the level of PSI core protein (PsaA) was analyzed. The level of PsaA increased in the initial three days of incubation, and declined gradually over time after five days of incubation (Figure 5).

**Figure 6:**
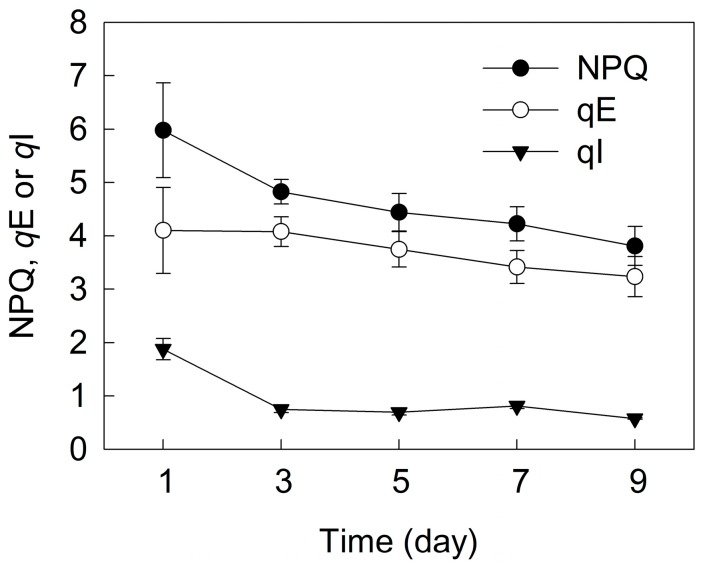
The NPQ (non-photochemical quenching), *q*E (fast component of NPQ) and *q*I (slow component of NPQ) during cell transformation and astaxanthin accumulation in *H. pluvialis* cultured in tubular photobioreactors. Mean ± SE of five replicates are presented.

Non-photochemical quenching (NPQ) in *H. pluvialis* decreased gradually during the incubation (Figure 6). The fast component of NPQ (*q*E) did not change in the initial three days of incubation, and decreased slightly over time after five days of incubation. The slow component of NPQ (*q*I) decreased sharply in the initial three days of incubation, and did not change over time after five days of incubation.

**Figure 7:**
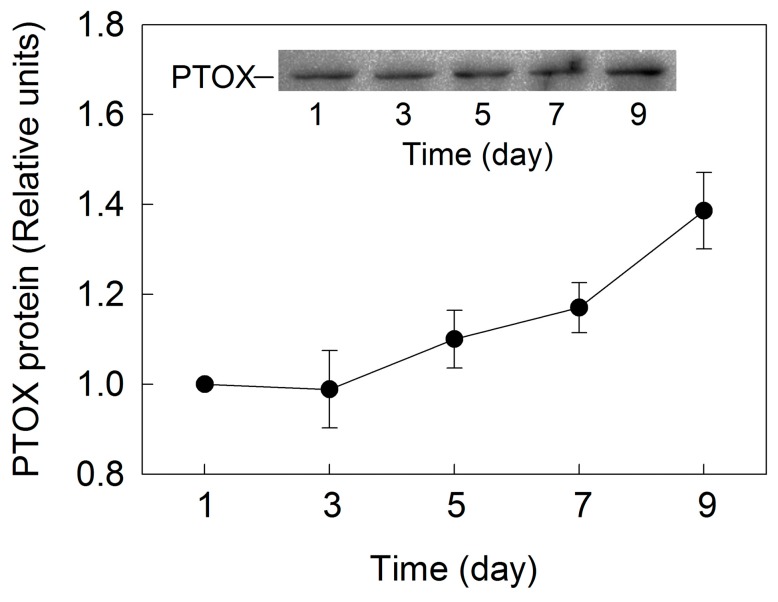
Densitometry of plastid terminal oxidase (PTOX) protein during cell transformation and astaxanthin accumulation in *H. pluvialis* cultured in tubular photobioreactors. Inset shows the levels of PTOX protein. Representative results of three independent replicates are shown.

Plastid terminal oxidase (PTOX) is the core protein of chlororespiration that oxidizes the plastoquinone (PQ) pool. The level of PTOX did not change in the initial three days of incubation, and increased gradually over time after five days of incubation in *H. pluvialis* (Figure 7).

**Figure 8:**
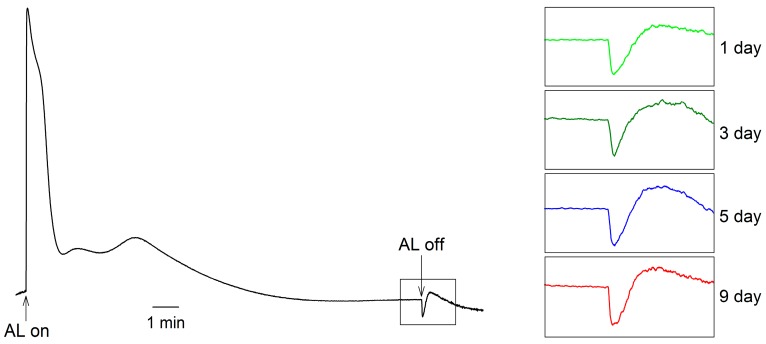
The postillumination increase in chlorophyll fluorescence during cell transformation and astaxanthin accumulation in *H. pluvialis* cultured in tubular photobioreactors. Insets show transient increase in chlorophyll fluorescence following light to dark transition. AL, white actinic light. Representative images from five independent replicates are shown in the figure.

A transient postillumination increase in chlorophyll fluorescence following light to dark transition is due to the reduction of PQ [23,24]. It shows that both the initial rate of the increasing phase and the amplitude of the postillumination increase in chlorophyll fluorescence in *H. pluvialis* did not change over time during the incubation (Figure 8).

**Figure 9:**
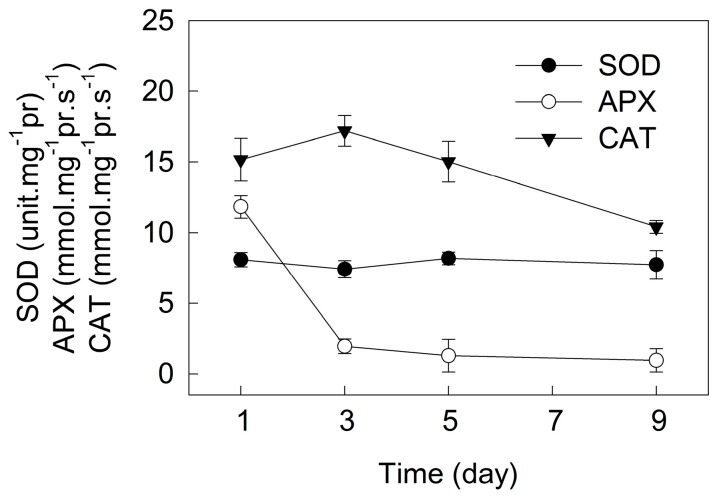
The activities of SOD (superoxide dismutase), APX (ascorbate peroxidase) and CAT (catalase) during cell transformation and astaxanthin accumulation in *H. pluvialis* cultured in tubular photobioreactors. Mean ± SE of five replicates are presented.

As shown in Figure 9, the superoxide dismutase (SOD) activity did not change over time during the incubation. The activity of ascorbate peroxidase (APX) decreased sharply in the initial three days of incubation, and did not change over time after five days of incubation. The activities of catalase (CAT) increased in the initial three days of incubation, and decreased gradually over time after five days of incubation.

**Figure 10:**
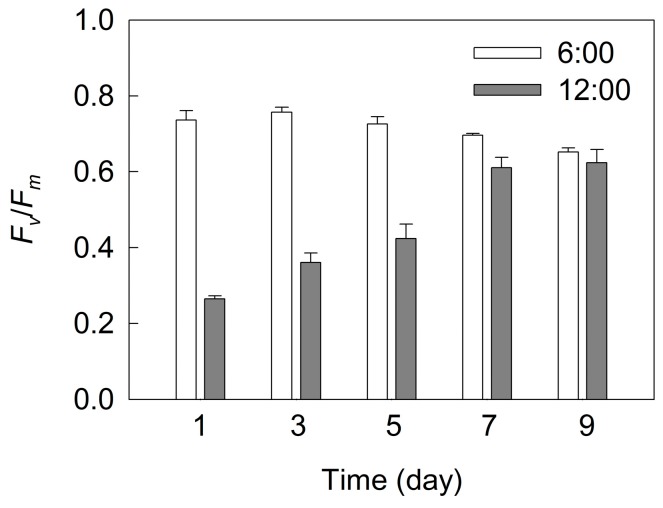
The maximal photochemical efficiency of photosystem II (*F_v_*/*F_m_*) during cell transformation and astaxanthin accumulation in *H. pluvialis* grown in tubular photobioreactors. Samples were taken at predawn (6:00) and at midday with photo flux density (PFD) approximately 2300 μmol·m^−2^·s^−1^ (12:00). Mean ± SE of five replicates are presented.

Maximal photochemical efficiency of PSII (*F_v_*/*F_m_*) is an indicator of photoinhibition in plants [25,26]. To examine the change in the capacity of photoprotection during the cell transformation and astaxanthin accumulation, we measured the change in *F_v_*/*F_m_* in *H. pluvialis* at predawn (6:00) and at midday with photo flux density (PFD) approximately 2300 μmol·m^−2^·s^−1^ (12:00) during the incubation (Figure 10). The *F_v_*/*F_m_* value measured at predawn was maintained at relatively stable values in the initial five days of incubation and decreased only slightly after seven days of incubation. However, the *F_v_*/*F_m_* measured at midday showed a considerable decrease before seven days of incubation, compared with that measured at predawn. The *F_v_*/*F_m_* measured at midday decreased by 64.0% in *H. pluvialis* cells at one day, but did not change in cells at nine days, compared with that measured at predawn (Figure 10).

## 3. Discussion

The green motile cells transformed into non-motile cells during the first three days of incubation (cell transformation phase), with noticeable amounts of astaxanthin having accumulated by five days of incubation (astaxanthin accumulation phase), in *H. pluvialis* grown outdoors in tubular photobioreactors (Figure 1). Chlorophyll content increased gradually with time during the initial seven days of incubation, but decreased slightly after seven days of incubation, which indicates that the nine-day incubation period in this experiment was the early stage of astaxanthin accumulation in *H. pluvialis* grown outdoors. We speculate that chlorophyll content will decrease significantly after nine days of incubation (late stage of astaxanthin accumulation).

The photosynthetic capacity increased during cell transformation, and then decreased gradually during astaxanthin accumulation (Figure 2). Meanwhile, multiple changes of photosynthetic behaviors occurred during cell transformation and astaxanthin accumulation (Figure 3). In detail, during the initial three days of incubation, there was relative variable fluorescence kinetics at the J-step, the *L*-band and the electron transport per active reaction center (ETo/RC) did not change, the *K*-band, the light absorption per active reaction center (ABS/RC) and the trapping of excitation energy per active reaction center (TRo/RC) decreased, whereas the maximal amplitudes of fluorescence in the I-P phase of the OJIP transient, the efficiency of electron transport (*ψ_o_*) and the quantum yield of electron transport (*φ_Eo_*) increased over time (Figure 3 and Figure 4). These results indicate that the light absorption and photosynthetic electron transport became much more efficient [17,22] during cell transformation, which was also indicated by the fact that the performance index PI_ABS_ as well as its individual partial components RC/ABS, *φ_Po_*/(1 − *φ_Po_*) and *ψ_o_*/(1 − *ψ_o_*) increased significantly during the initial three days of incubation (Figure 4).

After five days of incubation, during astaxanthin accumulation (Figure 1), the fact that the maximal amplitude in the I-P phase of the OJIP transient decreased obviously over time (Figure 3E) indicates a decrease in the pool size of PSI end electron acceptors, leading to an over-reduction of the PSI acceptor side [17,22,27]. Moreover, the relative variable fluorescence kinetics at the J-step increased with increased time after five days of incubation (Figure 3B), which indicates that the ratio of Q_A_^−^/Q_A_ increased [19,26], resulting in an over-reduction of the PSII acceptor side during astaxanthin accumulation.

The *L*-bands increased significantly over time after five days of incubation (Figure 3C), indicating that the PSII units were less grouped, resulting in a poorer utilization of the excitation energy and a lower stability of the system [17,26] during astaxanthin accumulation in *H. pluvialis*. The *K*-bands (Figure 3D) increased significantly with increased time, which suggests that the donor side of PSII might be impaired [15,17] during astaxanthin accumulation. Meanwhile, RC/ABS, *φ_Po_*/(1 − *φ_Po_*) and *ψ_o_*/(1 − *ψ_o_*) declined gradually during astaxanthin accumulation (Figure 4). All these results indicate that the capacity and efficiency of photosynthetic energy utilization decreased significantly during astaxanthin accumulation in *H. pluvialis*, leading to imbalance between photosynthetic light absorption and energy utilization. Moreover, the observation that ABS/RC increased but ETo/RC, *ψ_o_* and *φ_Eo_* decreased with increased time after five days of incubation (Figure 4) also indicates that the balance between photosynthetic light absorption and energy utilization was disturbed [17,22] during astaxanthin accumulation. The imbalance between light absorption and utilization will cause the over-excitation of PSII reaction centers [22,28].

The above results suggest that PSII activity increased during cell transformation phase but decreased gradually during the astaxanthin accumulation phase in *H. pluvialis* grown outdoors. The change of PsaA protein level (Figure 5) indicates that PSI activity also increased during the cell transformation phase but decreased gradually during astaxanthin accumulation phase in *H. pluvialis*. These results are consistent with a change of photosynthetic capacity (Figure 2).

Excess light energy will accelerate the generation of reactive oxygen species (ROS), leading to photoinhibition [22,29]. In order to escape from exposure to excess light, most plants have evolved various defence mechanisms to prevent the generation of ROS, such as NPQ [30,31], PTOX [32], PSI cyclic electron transport (CEF-PSI) [24,31] and antioxidant enzymes [33]. NPQ, of which the main component is *q*E, can dissipate excess absorbed light energy in PSII. The *q*E mechanism is controlled by the xanthophyll cycle. During cell transformation and astaxanthin accumulation in *H. pluvialis*, the photoprotective capacity of NPQ decreased gradually (Figure 6). Interestingly, NPQ value in this experiment is much higher (close to 6 at 1 day) than that in other reports [13,14], likely due to the difference of growth light intensities of *H. pluvialis*.

PTOX can remove electrons from the PQ pool, which may have a significant role in dissipation of excess electrons under high light [32]. PTOX activation is also correlated to the carotenoid accumulation [13]. The level of PTOX did not change during cell transformation phase, and increased gradually during astaxanthin accumulation phase (Figure 7), which is consistent with the process of astaxanthin synthesis during the incubation in *H. pluvialis* grown outdoors. However, the role of PTOX (photoprotection and/or activation of astaxanthin synthesis) in *H. pluvialis* during cell transformation and astaxanthin accumulation needs to be further investigated.

A transient postillumination increase in chlorophyll fluorescence following light to dark transition is caused by the reduction of PQ. This reaction is correlated to the balance between oxidation of PQ by PTOX and reduction of PQ by CEF-PSI [23]. According to the change of PTOX activity (Figure 7), both the initial rate of the increasing phase and the amplitude of the postillumination increase in chlorophyll fluorescence did not change over time during the incubation in *H. pluvialis* (Figure 8), indicating that CEF-I activity did not change during the cell transformation phase and increased gradually during the astaxanthin accumulation phase. The CEF-I might play an important role in photoprotection of *H. pluvialis* cells during astaxanthin accumulation.

Under photoinhibitory conditions (high light or UV light), there are two major sites of ROS generation in chloroplasts: the end of photosynthetic electron transport chain (acceptor side of PSI) and the PSII reaction centers [22,28,31,34]. The over-reduction of the PSI acceptor side and the over-excitation of PSII reaction centers during astaxanthin accumulation in *H. pluvialis* would inevitably enhance the generation of ROS, leading to more severe photoinhibition under high light. Meanwhile, the decrease in photosynthetic capacity during astaxanthin accumulation (Figure 2) also accelerated the generation of excess light energy. However, we compared the *F_v_*/*F_m_* value at predawn and at midday during the incubation of *H. pluvialis* (Figure 10). The results demonstrate that photoinhibition was more severe during cell transformation because of the decrease in photoprotective capacity, and the level of photoinhibition decreased significantly over time during astaxanthin accumulation, especially after seven days of incubation. This is reflected by the fact that *F_v_*/*F_m_* determined at midday decreased significantly during the initial three days of incubation, but was affected very little after seven days of incubation, compared with that determined at predawn (Figure 10). It has been suggested that astaxanthin can protect *H. pluvialis* cells against oxidative stress [35,36]. In our previous study, the role of two distinct antioxidative mechanisms, the defensive enzyme system and the astaxanthin were compared in *H. pluvialis* [37]. As an antioxidant, astaxanthin is more efficient than the defensive enzyme system. In the present study, SOD activity did not change over time during cell transformation and astaxanthin accumulation. The activity of APX decreased sharply during cell transformation, and did not change during astaxanthin accumulation. The activity of CAT increased during cell transformation and decreased gradually during astaxanthin accumulation (Figure 9). Therefore, in *H. pluvialis* grown outdoors, NPQ, PTOX, CEF-I, defensive enzymes (SOD, APX and CAT) activities and the accumulation of amounts of astaxanthin can protect cells against photoinhibition.

Moreover, it appears that in the initial three days of incubation (cell transformation phase), the high light at midday could lead to more severe photoinhibition and even decrease the cell numbers in cultures of green motile *H. pluvialis*. Therefore, in large-scale cultivation, the effective shade can protect green motile *H. pluvialis* cells against photoinhibition, thereby enhancing biomass for astaxanthin production.

## 4. Materials and Methods

### 4.1. Strains and Culture Conditions

The alga *Haematococcus pluvialis* (strain *H*_0_) was obtained from the Institute of Oceanology, Chinese Academy of Sciences, Qingdao, China. The seeding culture (the motile green vegetative cells) was prepared by using an exponentially growing culture of *H. pluvialis* grown outdoors in modified MCM medium (200 mg·L^−1^ NO_3_^−^) [37,38] in closed column photobioreactors, which was re-cultured in nitrogen-limited medium (50 mg·L^−1^ NO_3_^−^) up to a final concentration of 1.7 × 10^5^ cells·mL^−1^ in tubular photobioreactors (working volume 5000 L) outdoors in the afternoon (17:00). *H. pluvialis* cells incubated for 1, 3, 5, 7 and 9 days were used for all analyses. All of these experiments were done in the Yunnan Alphy Biotech Co., Ltd. (Chuxiong, China) facilities. During this period, there was typical Yunnan winter weather, with a mean daily air temperature between 17 and 21 °C and an average PFD of around 2300 μmol·m^−2^·s^−1^ at midday. The pigments, respiration rate, O_2_ evolution rate, PsaA protein level and chlorophyll *a* fluorescence (OJIP) transients were determined at predawn (6:00). The NPQ, PTOX protein level, postillumination increase in chlorophyll fluorescence and antioxidant enzyme (superoxide dismutase, ascorbate peroxidase and catalase) activities were determined at midday (12:00). The maximal photochemical efficiency of PSII (*F_v_*/*F_m_*) was determined at predawn and at midday (12:00).

### 4.2. Analytical Procedures

Algal astaxanthin was extracted with methanol/dichloromethane (1:3, *v*/*v*), and the extracts were analyzed by HPLC (Agilent, Santa Clara, CA, USA) following the protocol reported by Yuan et al. [39]. Algal chlorophyll was extracted with 80% acetone, and the extracts were analyzed with a UV-120 system (Shimadzu, Kyoto, Japan) according to the method of Porra [40].

The total photosynthetic O_2_ evolution rate and respiration rate were measured with a Clark-type O_2_ electrode (DW2, Hansatech, Norfolk, UK) according to Zhang and Liu [18].

Chlorophyll *a* fluorescence (OJIP) transients of cells during the incubation were measured with a Handy PEA fluorometer (Hansatech). All measurements were performed with 10 min dark-adapted cells. Fluorescence levels *F_o_* (50 μs), *F_K_* (300 μs), *F_J_* (2 ms), *F_I_* (30 ms), and *F_P_* (peak, about 300 ms) were recorded. The relative variable fluorescence kinetics (*W_OP_*, *W_OK_*, *W_OJ_*, and *W_OI_*) were obtained by normalizing the fluorescence transients according to the equations of the JIP-test [17,20,21]. Difference kinetics between the cells incubated for 1 d and other samples were calculated as: Δ*W* = *W* − *W*_(*cells incubated for 1 day*)_, where *W* was the relative variable fluorescence. In addition, the following parameters were used for the quantification of PSII behaviors [17,41]:

(1) The specific energy fluxes, i.e. the light absorption per active reaction center (ABS/RC, the average antenna size), the trapping of excitation energy per active reaction center (TRo/RC), the electron transport per active reaction center (ETo/RC).
(2) The flux ratios or yields, i.e., the efficiency of electron transport (*ψ_o_*), and the quantum yield of electron transport (*φ_Eo_*).
(3) The photosynthetic performance index PI_ABS_ as well as its individual partial components, the efficiency of light absorption (RC/ABS), the performance due to the quantum efficiency of primary photochemistry (*φ_Po_*/(1 − *φ_Po_*)) and the performance due to the quantum efficiency of the conversion of excitation energy to electron transport (*ψ_o_*/(1 − *ψ_o_*)), were also calculated according to the JIP-test equations.

The NPQ were measured with a FMS-2 pulse-modulated fluorometer (Hansatech, Norfolk, UK) as described in Jiang et al. [42]. Components of NPQ, including a fast component (*q*E) and a slow component (*q*I), were determined following the protocol of Johnson et al. [43].

The PsaA and PTOX protein was detected by Western Blot according to Fan et al. [44]. The total proteins were denatured and separated using a 12% polyacrylamide gradient gel. The denatured protein complexes in the gel were then electro-blotted to polyvinylidene fluoride (PVDF) membranes, probed with PsaA or PTOX antibody, and then visualized by the enhanced chemiluminescence method. The quantitative image analysis of protein levels was performed with Gel-Pro Analyzer 4.0 software (Media Cybernetics, Bethesda, MD, USA).

The postillumination increase in chlorophyll fluorescence was measured with an FMS-2 pulse-modulated fluorometer (Hansatech, Norfolk, UK) according to Wang et al. [24].

The activities of superoxide dismutase (SOD; EC 1.15.1.1), ascorbate peroxidase (APX; EC 1.11.1.11) and catalase (CAT; EC 1.11.1.6) were determined according to Jia et al. [45].

Maximal photochemical efficiency of PSII (*F_v_*/*F_m_*) of cultures during incubation was measured using a Handy PEA fluorometer (Hansatech, Norfolk, UK) [26]. *F_v_*/*F_m_* measurement was performed using cultures that had been dark-adapted for 10 min prior to doing the measurement.

Least significant difference (LSD) was used to analyze differences between the measurements.

## 5. Conclusions

In *H. pluvialis* grown outdoors in tubular photobioreactors, during the cell transformation phase, the light absorption and photosynthetic electron transport became much more efficient, and the capacities of photoprotective mechanisms decreased. During astaxanthin accumulation, the pool size of PSI end electron acceptors decreased significantly, leading to an over-reduction of the PSI acceptor side. The balance between photosynthetic light absorption and energy utilization was disturbed, resulting in the over-excitation of PSII reaction centers. The over-reduction of the PSI acceptor side and the over-excitation of the PSII reaction centers during astaxanthin accumulation would inevitably enhance the generation of ROS, leading to more severe photoinhibition under high light, e.g., at midday. However, the capacity for photoprotection in *H. pluvialis* increased significantly with time during astaxanthin accumulation, which might be because the NPQ, PTOX, CEF-I, defensive enzyme (SOD, APX and CAT) activities and the accumulated astaxanthin can protect cells against photoinhibition.
